# GeneXpert MTB/RIF Ultra vs Unstimulated Interferon γ (IRISA-TB) for the Diagnosis of Tuberculous Pericarditis in a Tuberculosis-Endemic Setting

**DOI:** 10.1093/ofid/ofae021

**Published:** 2024-03-20

**Authors:** Philippa Randall, Aliasgar Esmail, Lindsay Wilson, Edson Makambwa, Anil Pooran, Michele Tomasicchio, Keertan Dheda, Mpiko Ntsekhe

**Affiliations:** Division of Pulmonology, Centre for Lung Infection and Immunity, Department of Medicine, University of Cape Town, Observatory, South Africa; Antrum Biotech, Observatory, South Africa; Division of Pulmonology, Centre for Lung Infection and Immunity, Department of Medicine, University of Cape Town, Observatory, South Africa; Division of Pulmonology, Centre for Lung Infection and Immunity, Department of Medicine, University of Cape Town, Observatory, South Africa; Division of Pulmonology, Centre for Lung Infection and Immunity, Department of Medicine, University of Cape Town, Observatory, South Africa; Division of Pulmonology, Centre for Lung Infection and Immunity, Department of Medicine, University of Cape Town, Observatory, South Africa; Division of Pulmonology, Centre for Lung Infection and Immunity, Department of Medicine, University of Cape Town, Observatory, South Africa; Division of Pulmonology, Centre for Lung Infection and Immunity, Department of Medicine, University of Cape Town, Observatory, South Africa; Department of Infection Biology, Faculty of Infectious and Tropical Diseases, London School of Hygiene and Tropical Medicine, London, UK; Division of Cardiology, Department of Medicine, University of Cape Town, Observatory, South Africa

**Keywords:** accuracy, diagnosis, GeneXpert Ultra, interferon γ, tuberculous pericarditis

## Abstract

**Background:**

Tuberculous pericarditis (TBP) is a paucibacillary disease, where host biomarkers such as unstimulated interferon γ (IRISA-TB) have high diagnostic accuracy. However, DNA-based diagnostic tests (GeneXpert Ultra), more sensitive than an earlier versions, have recently become available. Given that the diagnosis of TBP is challenging, we performed a comparative diagnostic accuracy study comparing both assays.

**Methods:**

We recruited 99 consecutive patients with suspected TBP in Cape Town, South Africa. Definite TBP was defined by microbiological confirmation of tuberculosis (TB) on pericardial fluid culture or an alternative polymerase chain reaction–based test (GeneXpert MTB/RIF) or by use of sputum (polymerase chain reaction or culture). Probable TBP was defined as a high clinical suspicion of TB accompanied by anti-TB treatment, while non-TBP was defined as negative microbiological test results for TB without initiation of TB treatment and/or the presence of an alternative diagnosis.

**Results:**

There were 39 patients with definite TBP, 35 with probable TBP, and 23 with non-TBP. Approximately 70% of participants who received TB treatment were HIV coinfected. Overall, IRISA-TB was more sensitive than Xpert Ultra (88.6% [95% CI, 74.1%–95.5%] vs 71.5% [55.0%–83.7%], n = 53) and significantly more sensitive in participants who were HIV uninfected (100% [95% CI, 72.3%–100.0%] vs 60% [31.3%–83.2%], *P* = .03). In patients with definite and probable TBP combined (n = 84), sensitivity was significantly higher with IRISA-TB (77.3% [95% CI, 65.9%–85.8%] vs 37.9 [27.2%–50.0%], *P* < .0001). A similar pattern was seen in persons who were HIV uninfected (88.3% vs 35.3%, *P* = .002). Specificity was high for both assays (>95%).

**Conclusions:**

Unstimulated interferon γ (IRISA-TB) was significantly more sensitive than Xpert Ultra for the diagnosis of TB pericarditis in a TB-endemic resource-poor setting.

Globally, tuberculosis (TB) remains out of control with an estimated ∼11 million incident cases of TB and 1.5 million deaths in 2022 [1]. Among those who were newly ill, ∼15% to 20% had extrapulmonary TB (EPTB). TB serositis (specifically pleural TB) is one of the most common forms of EPTB globally and often the dominant form of EPTB seen in TB-endemic countries [2]. Although tuberculous pericarditis (TBP) makes up a smaller proportion of TB serositis and EPTB, the associated 6-month mortality remains high (20%–40%), accounting for between 5% and 10% of hospital admissions for acute heart failure in Africa [3]. Despite the significant burden of disease and the high mortality, the diagnosis of TBP remains challenging. Smear microscopy and culture have a sensitivity of ∼5% and 50%, respectively [4]. Alternative approaches to making a diagnosis of TBP that integrate pericardial fluid biochemical tests (eg, adenosine deaminase), clinical scoring tools (eg, Tygerberg diagnostic index), and DNA-based diagnostic tests (eg, GeneXpert MTB/RIF) also perform poorly [5, 6]. Although Xpert MTB/RIF is endorsed by the World Health Organization as a first-line test in many forms for TB, studies focused on its accuracy in TBP have been limited by small numbers of patients [7–10], and in a recent meta-analysis that used culture and pericardial biopsy as a reference standard, its sensitivity was reported to be suboptimal (<60%) [11].

More recently, the manufacturer Cepheid has developed a cartridge for the detection of TB, designated the GeneXpert MTB/RIF Ultra [12]. When compared with the GeneXpert MTB/RIF assay, this assay was ∼5% more sensitive in smear-positive pulmonary TB and 17% more sensitive in smear-negative pulmonary TB [12], and the level of detection in sputum improved from ∼115 to ∼15 colony-forming units (CFU)/mL [13]. Given these considerations and the absence of published data about the performance of Xpert Ultra in TB pericarditis (TBP), we performed a prospective study evaluating Xpert Ultra against a host biomarker, unstimulated interferon γ (IFNγ; IRISA-TB [Antrum Biotech]). Unstimulated IFNγ was chosen as a comparator because it has been shown to be a promising biomarker for the diagnosis of TB serositis in general [8, 14, 15] and for the diagnosis TBP specifically [16]. This is in contradistinction to IFNγ release assays, which require overnight stimulation and are not recommended by the World Health Organization for diagnosis of TBP [17]. Indeed, a recent meta-analysis of studies assessing the diagnostic accuracy of unstimulated IFNγ for TBP confirmed its high diagnostic accuracy with a pooled sensitivity and specificity of 0.97 (95% CI, .87–.99) and 0.99 (95% CI, .74–1.00), respectively [18].

## METHODS

### Patient Recruitment, Categorization, and Routine Laboratory Testing

Patients with suspected pericardial TB (large pericardial effusion; defined as echo-free space around the heart >1 cm in diastole) were prospectively recruited from Groote Schuur Hospital in Cape Town, South Africa. The University of Cape Town Human Research Ethics Committee approved the study (HREC 370/2015). All patients provided informed consent for study participation. Pericardial fluid was collected by ultrasound-guided pericardiocentesis. Pericardial fluid samples were subjected to routine biochemical and cytologic analysis by the National Health Laboratory Services. This included protein, albumin, adenosine deaminase (cutoff, 30 IU/L), glucose, cell differentials, cytology, concentrated smear microscopy, and liquid culture for *Mycobacterium tuberculosis* with the MGIT 960 (Becton, Dickinson). The remaining fluid was placed in a biobank, frozen at −20 °C, and subsequently used for Xpert Ultra and IRISA-TB analyses. HIV testing was performed in consenting patients.

Due to the limitations of a single pericardial fluid TB culture for confirming a diagnosis (∼40% sensitivity), a composite micropathologic reference standard was used for patient categorization (this reference standard was used in all analyses presented). Patients were categorized as follows:

*Definite TB:* patients with 1 positive *M tuberculosis* culture result (pericardial fluid, biopsy specimen, and/or sputum) and/or caseating granulomatous inflammation suggestive of TB on histologic examination and improvement with anti-TB treatment. All patients in this category received anti-TB treatment.

*Probable TB:* patients not meeting the micropathologic criteria for definite TB but who had clinical indicators suggestive of TB and who were administered anti-TB treatment based on high clinical suspicion of having TB. All patients in this category received anti-TB treatment based on clinical and laboratory criteria.

*Non-TB:* patients who did not fit the criteria for definite and probable TB, with no microbiological or histological evidence of *M tuberculosis* and/or for whom an alternative diagnosis was available. These patients did not receive anti-TB treatment at presentation or follow-up.

Patients who did not fall into any of these categories remained unclassified.

### Unstimulated IFNγ Measurement: IRISA-TB

Of the 97 enrolled participants, 84 had sufficient pericardial fluid to measure IFNγ concentrations with the IRISA-TB assay according to the manufacturer's instructions. The assay was performed in duplicate and the average values reported. Pericardial fluid supernatant was prepared by centrifuging 1 mL of pleural fluid at 12 000 relative centrifugal force for 90 seconds.

### Cepheid GeneXpert MTB/RIF and GeneXpert Ultra Assays

The Xpert Ultra and Xpert MTB/RIF assays were performed with 1 mL of pericardial fluid diluted with 2 mL of Xpert sample buffer, followed by vigorous mixing. Xpert Ultra and Xpert MTB/RIF cartridges were run on a GeneXpert 4-module machine (Dx System version 4.7b; Cepheid). Of the 97 enrolled participants, Xpert Ultra was performed on the pericardial fluid of 84.

### Definitions and Micropathologic Reference Standard Used in This Study

#### Definite TB Pericardial Effusion

The presence of any 1 or more of the following criteria:

- Microbiologically confirmed pericardial effusion (Xpert and/or smear and/or culture)
- The presence of necrotizing granulomatous inflammation (with or without acid-fast bacilli) on pathologic evaluation of fluid (cytology) or pericardial tissue sample (where available)
- Concurrent microbiologically confirmed sputum for TB (Xpert and/or culture) within 4 weeks (28 days) of sampling the pericardial effusion

#### Non–TB Pericardial Effusion

All of the following criteria:

- Microbiologically negative pericardial effusion for TB (Xpert and smear and culture)
- Absence of necrotizing granulomatous inflammation on pathologic evaluation of fluid or tissue (where available), with or without a suggestion for an alternative diagnosis
- Patient not prescribed TB treatment

#### Probable Pericardial Effusion

All of the following criteria:

- Patient presenting with a clinical syndrome suggestive of chronic pericardial effusion (clinically suspected TBP)
- Patients not meeting criteria outlined for “definite”
- Empiric TB treatment initiation by the clinician based on suspicion of TB
- Documented clinical response to TB treatment at 2- to 4-month follow-up in the opinion of the treating clinician
- Absence of a more plausible alternative diagnosis for TBP

#### Unclassifiable

- Patients not falling into the defined 3 groups (eg, patients with nondiagnostic pericardial fluid sample who were lost to follow-up or who died before treatment could be initiated)

### Sample Size Estimations

By assuming a sensitivity for TBP of ∼60% for Xpert Ultra [19] and ∼95% for unstimulated IFNγ [18] and at 80% power and 95% confidence, ∼30 patients with definite TB would be required to determine a statistically significant difference between Xpert Ultra and IRISA-TB after accounting for a 5% loss to follow-up.

### Statistical Analysis

Diagnostic accuracy, including 95% CIs, was assessed with sensitivity, specificity, positive predictive value (PPV), negative predictive value (NPV), and area under the receiver operator characteristic curve in the definite TB and non-TB groups. Unpaired and paired categorical variables were compared with χ^2^ and McNemar tests, respectively. Continuous variables were compared with a Student *t* test where appropriate. The Mann-Whitney and Wilcoxon rank sum test was used for unpaired and paired nonparametric continuous variables. Statistical analyses were performed with Prism (version 6.0; GraphPad), MedCalc (version 18.6), and Microsoft Excel.

## RESULTS

### Clinical and Demographic Data

A total of 99 patients were recruited into the study: 39 had definite TB, 23 were classified as non-TB, and 35 as probable TB. Those with non-TB effusions had a spectrum of malignant and nonmalignant diagnoses, including lymphoma, adenocarcinoma, bacterial pericarditis, and hypothyroidism. Two patients had insufficient clinical data to be categorized in the previously described groups and were subsequently excluded.

Definite and probable TB groups had higher HIV infection rates and New York Heart Association class I–II and III–IV vs the non-TB group (*P* < .0001, .0031, and .0031, respectively). Diastolic blood pressure, whole cell counts (serum), and creatinine levels were significantly lower in the definite and probable TB groups (*P =* .0237, .0048, and .0480).

**Figure 1.**
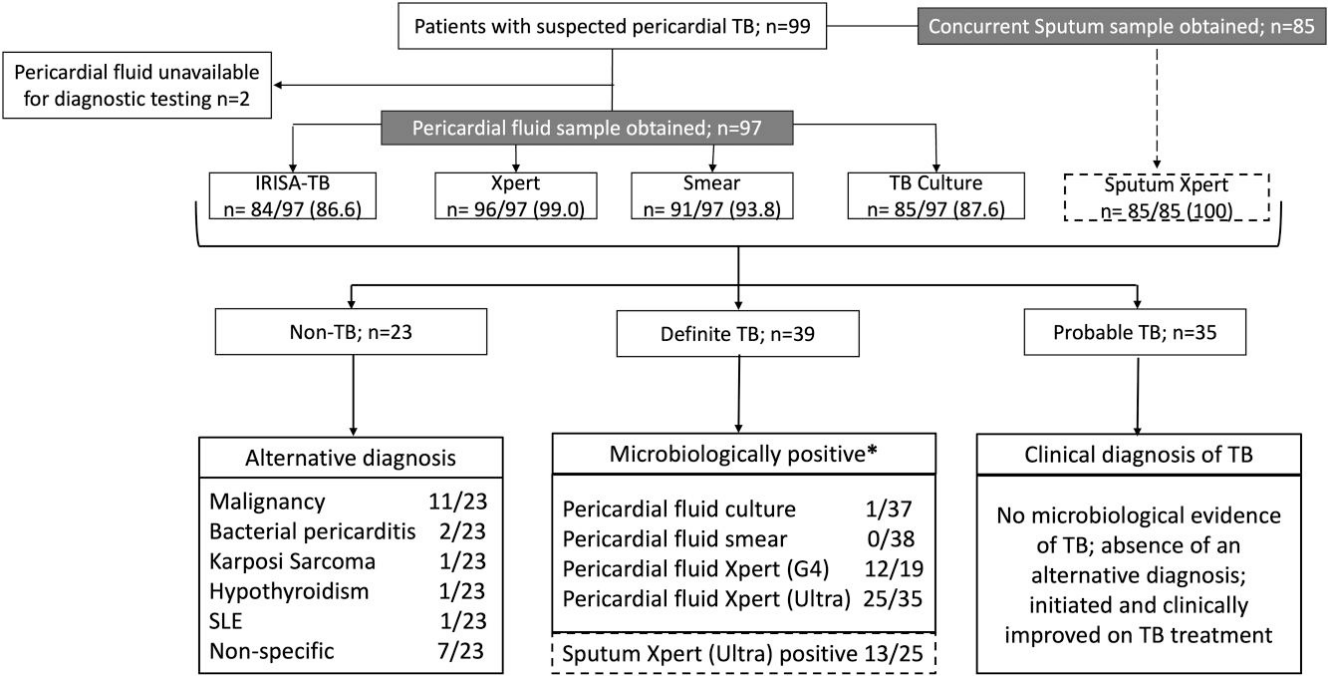
Study overview of patient groups, investigations performed, and tests undertaken. *Patients tested positive on multiple diagnostic tests, causing the tally of positive test results to exceed the number of patients in this group. SLE, systemic lupus erythematosus; TB, tuberculosis.

A study overview is provided in Figure 1. Demographic and clinical data are summarized in Table 1.

**Table 1.** Baseline Characteristics of the Definite, Probable, and Non-TB Groups: Demographic, Clinical, Serum, Electrocardiograph, and Pericardial Fluid Analysis

	No. (%) or Median (IQR)					
	Definite TB (n = 39)	Non-TB (n = 23)	*P* Value^a^	Probable TB (n = 35)	Definite and Probable TB (n = 74)	*P* Value^b^
Male	23 (59)	11 (47.8)		23 (65.7)	46 (62.2)	
Age, y	41 (34–50)	58 (51–63)	**.0004**	38 (31–48)	40 (33–49)	**<.0001**
HIV						
Infected	27 (69.2)	5 (21.7)	**.0003**	27 (77.1)	54 (73)	**<.0001**
Uninfected	12 (30.8)	18 (78.3)	**.0003**	8 (22.9)	20 (27)	**<.0001**
CD4 count, cells/mL	129.5 (77–187.5)	168 (100.5–382)		176 (122–381)	158 (83.5–222)	
ARV therapy	10 (37)	3 (60)		15 (55.6)	25 (33.8)	
Previous TB	11 (28.2)	5 (21.7)		9 (25.7)	20 (27)	
NYHA class						
I–II	24 (61.5)^c^	9 (39.1)		30 (85.7)	54 (73)	**.0031**
III–IV	15 (38.5)^c^	14 (60.9)		5 (14.3)	20 (27)	**.0031**
Pulse, beat/min	110 (102–125)	103 (90–130)		106 (100–120)	108.5 (100–123)	
Blood pressure, mm Hg						
Systolic	113 (105–121)	121 (112–129)		115 (107–127)	114 (106–124)	
Diastolic	70 (61–77)	77 (68–90)	**.0241**	70 (66–80)	70 (61.75–78.25)	**.0237**
Serum						
Total WCC, X109/L	6.32 (4.58–8.39)	9.96 (7.04–15.98)	**.0016**	6.41 (4.71–8.79)	6.39 (4.65–8.65)	**.0048**
Creatinine, μmol/L	66.5 (54.5–83.75)^d^	74 (62–98.5)		61 (49–76)	64 (52–82)	**.048**
Hemoglobin, g/L	10.75 (9.2–11.45)	10.55 (9.58–12.23)		9.4 (8.6–11.2)	10.3 (8.8–11.3)	
Effusion						
Global	35 (89.8)	20 (87)		28 (80)	63 (85.1)	
Other	2 (5.1)	0		4 (11.4)	6 (8.1)	
Unknown	2 (5.1)	3 (13)		3 (8.6)	5 (6.8)	
Tamponade	27 (69.2)	15 (65.2)		23 (65.7)	50 (67.6)	
ADA, IU	56.65 (49.88–78.15)	22.2 (15.08–26.5)	**<.0001**	43.15 (35.98–54.53)	51.85 (40.05–65.93)	**.0001**
Total protein, g/L	59 (51–70)	59 (50.5–65.75)		66 (58–70.25)	65 (52–70)	
LDH, IU	970 (642–1599)	1039 (283–1996)		603 (456–703.3)	708 (499–1050)	
Count						
Lymphocyte	1.26 (0.54–2.27)	1.88 (0.56–3.52)		1.51 (0.68–2.6)	1.37 (0.6–2.45)	
Neutrophil	0.63 (0.47–1.22)	0.99 (0.09–1.67)		0.39 (0.15–1.48)	0.59 (0.26–1.29)	
IFNγ, pg/mL	93.7 (26.1–325.1)	0 (0–0)	**<.0001**	54.6 (0–150.4)	62 (10.43–223.6)	**<.0001**
TTP, d	12 (6–13)	…		…	12 (6–13)	
CT, unit	26 (24–28)	…		…	26 (24–28)	

Continuous data were analyzed by unpaired *t* test and categorical data by χ^2^ test. Only significant *P* values are shown (*P* < .05). Bold values in table denotes significant *P* values.

Abbreviations: ADA, adenosine deaminase; ARV, antiretrovirals; CT, cycles threshold; IFNγ, interferon γ; LDH, lactate dehydrogenase; NYHA, New York Heart Association; TB, tuberculosis; TTP, time to positive culture (days); WCC, white cell count.

^a^Definite vs non-TB.

^b^Definite and probable vs non-TB.

^c^ *P* = .0903 vs non-TB.

^d^ *P* = .0671 vs non-TB.

### Performance Outcomes of IRISA-TB: Definite and Non-TB

**Figure 2.**
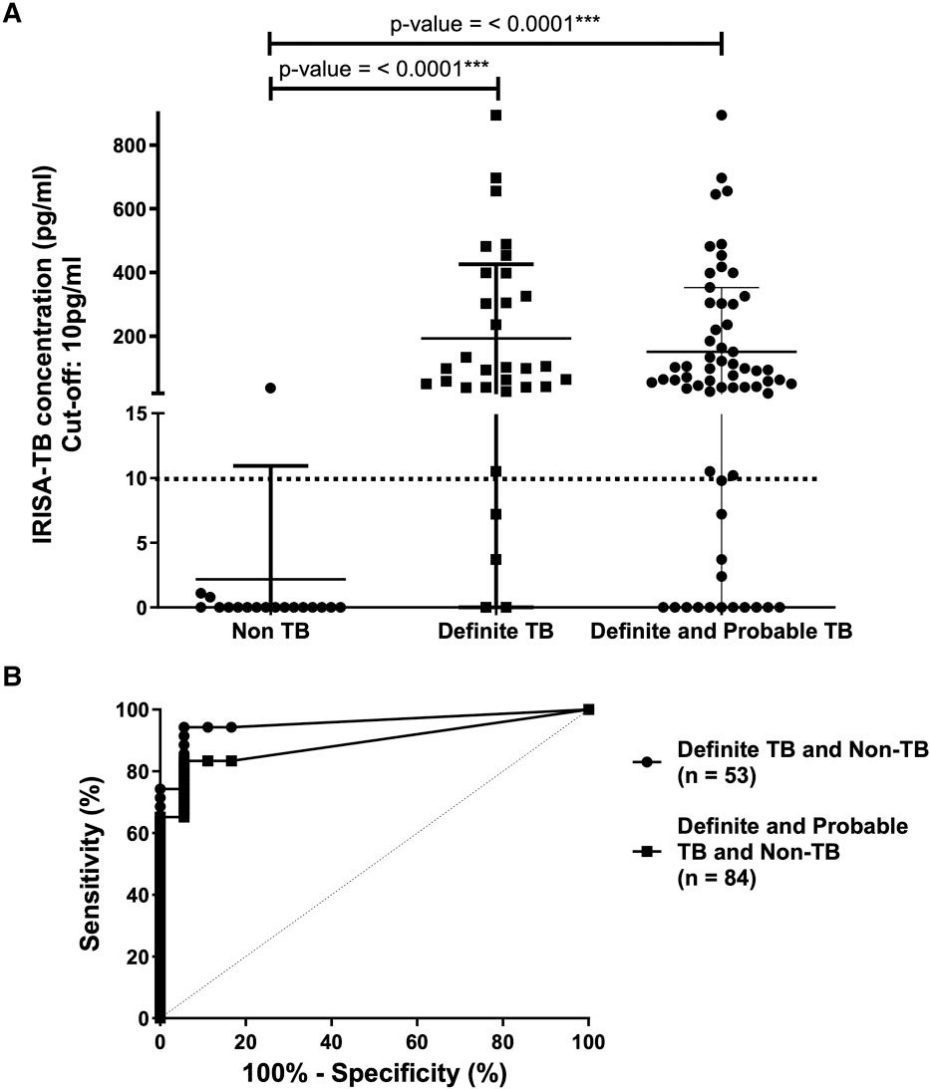
Performance of IRISA-TB in differentiating definite TB and/or probable TB from non-TB according to pericardial fluid from patients with suspected pericardial TB. *A*, IFNγ levels measured by IRISA-TB; *P* value by Mann-Whitney test. ROC-derived cut point of 10 pg/mL of IFNγ for IRISA-TB is indicated by dotted line. Data are presented as median (IQR). *B*, Area under the ROC curves for IRISA-TB based on the definite TB and non-TB groups (circles) and the definite and probable TB groups and the non-TB group (squares). Areas under the curve were 0.96 (definite TB vs non-TB) and 0.89 (definite and probable TB vs non-TB). No significant difference was observed between the ROC curves. IFNγ, interferon γ; ROC, receiver operator characteristic; TB, tuberculosis.

IFNγ levels (n = 53; 9 patients did not have remnant pericardial fluid for evaluation) were significantly higher in definite TB than non–TB pericardial effusions (median, 93.7 pg/mL [IQR, 26.1–325.1] vs 0.0 [0.0–0.0]; *P* < .0001; Figure 2*A*). Based on the definite and non-TB groups, a receiver operating characteristic curve derived a rule-in cut point of 10 pg/mL. Sensitivity, specificity, PPV, and NPV of IRISA-TB were 88.6% (95% CI, 74.1%–95.5%), 94.5% (74.3%–99.1%), 96.9% (84.3%–99.5%), and 81% (60%–92.4%), respectively (Figure 2*B*). Table 2 compares the diagnostic accuracy of IRISA-TB with Xpert Ultra assay in the definite vs non-TB groups. The sensitivity of IRISA-TB was superior to Xpert Ultra MTB/RIF assay in individuals infected with HIV (100% vs 60%, *P* = .0293).

**Table 2.** Accuracy of Xpert Ultra and IRISA-TB for the Diagnosis of Pericardial TB in Definite and Non-TB Cases

Definite TB vs Non-TB	Sensitivity, %	Specificity, %	PPV, %	NPV, %	AUC	PLR	NLR	DOR	NNT
IRISA-TB (cutoff, 10 pg/mL)									
Test performance: overall (n = 53)	88.6^a^ (74.1–95.5)	94.5 (74.3–99.1)	96.9 (84.3–99.5)	81 (60–92.4)	0.96 (0.90–1.01)	15.94	0.121	131.8 (13.61–1276)	3.1
No.	31/35	17/18	31/32	17/21					
Test performance: HIV (n = 30)	84 (65.4–93.6)	80 (37.6–96.4)	95.5 (78.3–99.2)	50 (21.6–78.5	0.88 (0.74–1.02)	4.20	0.200	21 (1.83–240.7)	2.8
No.	21/25	4/5	21/22	4/8					
Test performance: no HIV (n = 23)	100^b^ (72.3–100)	100 (77.2–100)	100 (72.3–100)	100^a^ (77.2–100)	1.00 (1.00–1.00)	…	0	567 (10.35–31051)	3.8
No.	10/10	13/13	10/10	13/13					
Xpert Ultra									
Test performance: overall (n = 53)	71.5^a^ (55–83.7)	100 (82.5–100)	100 (86.7–100)	64.3 (45.9–79.3)	…	…	0.285	89.86 (4.94–1633)	3.9
No.	25/35	18/18	25/25	18/28					
Test performance: HIV (n = 30)	76 (56.6–88.6)	100 (56.6–100)	100 (83.2–100)	45.5 (21.3–72)	…	…	0.24	33 (1.60–682.2)	3.1
No.	19/25	5/5	19/19	5/11					
Test performance: no HIV (n = 23)	60^b^ (31.3–83.2)	100 (77.2–100)	100 (61–100)	76.5^a^ (52.8–90.5)	…	…	0.40	39.00 (1.81–839)	6.3
No.	6/10	13/13	6/6	13/17					
*P* value	.0755,^a^ .0293^b^			.0649^a^					

The reference for definite TB was positive *Mycobacterium tuberculosis* pericardial fluid and/or sputum culture and/or histology in keeping with *M tuberculosis* infection. Non-TB was defined as no microbiological or histological evidence of *M tuberculosis* and/or an alternative diagnosis being available. NNT (cohort/number of positive test results) denotes the number of individuals who need to be tested before one identifies a positive case. Superscript letters denote comparisons for *P* values. Data in parentheses indicate 95% CI.

Abbreviations: AUC, area under the curve; DOR, diagnostic odds ratio; NLR, negative likelihood ratio; NNT, number needed to test; NPV, negative predictive value; PLR, postive likelihood ratio; PPV, positive predictive value; TB, tuberculosis.

### Performance Outcomes of IRISA-TB: Definite and Probable TB and Non-TB

IFNγ levels (n = 84) were significantly higher in definite and probable TB than non–TB pericardial effusions (median, 62 pg/mL [IQR, 10.43–223.6] vs 0.0 [0.0–0.0]; *P* < .0001; Figure 2*A*). According to the definite, probable, and non-TB groups at a rule-in cut point of 10 pg/mL, sensitivity, specificity, PPV, and NPV of IRISA-TB were as follows: 77.3% (65.9%–85.8%), 94.5% (74.3%–99.1%), 98.1% (89.9%–99.7%), and 53.2% (36.5%–69.2%), respectively (Figure 2*B*). Table 3 compares the diagnostic accuracy of IRISA-TB with Xpert Ultra in the definite and probable TB vs non-TB groups. The sensitivity of IRISA-TB was superior in comparison with Xpert (77.3% vs 37.9%, *P* < .0001), and this significance was carried through to individuals who were HIV infected (73.5% vs 38.8%, *P* = .0006) and uninfected (88.3% vs 35.3%, *P* = .0017). In addition, the NPV of IRISA-TB was significantly better than that of Xpert Ultra (53.2% vs 30.6%, *P* = .0431), and this significance was also observed in persons uninfected by HIV (86.7% vs 54.2%, *P* = .0386).

**Table 3.** Accuracy of Xpert Ultra and IRISA-TB for the Diagnosis of Pericardial TB in Definite and Probable vs Non-TB Cases

Definite and Probable TB vs Non-TB	Sensitivity, %	Specificity, %	PPV, %	NPV, %	AUC	PLR	NLR	DOR
IRISA-TB (cutoff, 10 pg/mL)								
Test performance: overall (n = 84)	77.3^a^ (65.9–85.8)	94.5 (74.3–99.1)	98.1 (89.9–99.7)	53.2^a^ (36.5–69.2)	0.89 (0.82–0.96)	13.91	0.240	57.8 (7.09–471)
No.	51/66	17/18	51/52	17/32				
Test performance: HIV (n = 54)	73.5^b^ (59.8–83.8)	80 (37.6–96.4)	97.3 (86.2–99.6)	23.6 (9.6–47.3)	0.81 (0.67–0.95)	3.67	0.331	11.08 (1.13–108.5)
No.	36/49	4/5	36/37	4/17				
Test performance: no HIV (n = 30)	88.3^c^ (65.7–96.8)	100 (77.2–100)	100 (79.7–100)	86.7^b^ (62.2–96.3)	0.94 (0.85–1.04)	…	0.117	167.4 (7.37–3805)
No.	15/17	13/13	15/15	13/15				
Xpert Ultra								
Test performance: overall (n = 84)	37.9^a^ (27.2–50)	100 (82.5–100)	100 (86.7–100)	30.6^a^ (20.3–43.2)	…	…	0.621	22.73 (1.31–394.1)
No.	25/66	18/18	25/25	18/59				
Test performance: HIV (n = 54)	38.8^b^ (26.5–52.8)	100 (56.6–100)	100 (83.2–100)	14.3 (6.3–29.4)	…	…	0.612	7.03 (0.37–134.5)
No.	19/49	5/5	19/19	5/35				
Test performance: no HIV (n = 30)	35.3^c^ (17.4–58.7)	100 (77.2–100)	100 (61–100)	54.2^b^ (35.1–72.2)	…	…	0.647	15.26 (0.77–301.3)
No.	6/17	13/13	6/6	13/24				
*P* value	<.0001,^a^ .0006,^b^ .0017^c^			.0431,^a^ .0386^b^				

Superscript letters denote comparisons for *P* values. Data in parentheses indicate 95% CI.

Abbreviations: AUC, area under the curve; DOR, diagnostic odds ratio; NLR, negative likelihood ratio; NPV, negative predictive value; PLR, positive likelihood ratio; PPV, positive predictive value; TB, tuberculosis.

### Performance Outcome of Xpert Ultra Assay

The sensitivity, specificity, PPV, and NPV of Xpert Ultra (n = 53) in definite and non-TB was 71.5% (95% CI, 55%–83.7%), 100% (82.5%–100%), 100% (86.7%–100%), and 64.3% (45.9%–79.3%), respectively (Table 2). The sensitivity, specificity, PPV, and NPV of Xpert Ultra (n = 84) in definite, probable, and non-TB was 37.9% (95% CI, 27.2%–50%), 100% (82.5%–100%), 100% (86.7%–100%), and 30.6% (20.3%–43.2%; Table 3).

## DISCUSSION

There are 2 key findings from this study. First, unstimulated IFNγ (IRISA-TB) had superior performance to Xpert Ultra in patients with definite TB and definite/probable TB combined when compared with those with non-TBP. Second, despite its improved sensitivity relative to other nucleic acid amplification tests, the NPV for Xpert Ultra remained very low.

Although Xpert Ultra is more sensitive than Xpert MTB/RIF, the chronic and paucibacillary nature of the disease is below the detection limit of the assay. This is not surprising given that the sensitivity of culture for confirmed TBP is only ∼30% to 50% [4]. Thus, in patients with TBP and other paucibacillary forms of TB, such as TB meningitis and TB serositis (pleural, peritoneal TB), conventional microbiological tests such as culture and nucleic acid amplification tests often have suboptimal sensitivity. We previously showed that lipoarabinomannan (LAM) antigen levels in the pericardium were low and had poor sensitivity for the diagnosis of a TB etiology [8]. Nevertheless, in this and other compartments, there are potent Th-1 proinflammatory responses with physiologic “trapping” of IFNγ, the mechanics and pathophysiology of which are poorly understood. Indeed, several studies have now shown the superior utility of host biomarkers in TB serositis and TB meningitis [9, 14, 15, 20]. We have demonstrated for the first time that despite the higher sensitivity of Xpert Ultra as compared with Xpert MTB/RIF, it still fails to outperform host biomarkers for this category of disease and may be too low to reliably rule out TBP in practice. Yet, IRISA-TB is now available as a single- or multiple-use test marketed by a South African–based company (https://antrumbiotech.co/innovations/), and we performed an independent evaluation of this assay.

Diagnostic evaluation in the face of an imperfect reference standard is tricky. Given that conventional diagnostics, including culture and Xpert MTB/RIF, have sensitivities of only 50% to 60%, in the absence of pericardial biopsy, inferring a definitive diagnosis of TB is challenging. To maximize the accuracy of our reference standard, we used a combination of culture and, to avoid confirmation bias, an alternative nucleic acid amplification test (ie, Xpert MTB/RIF) as compared with the one being evaluated (Xpert Ultra). However, given the inadequacy of existing microbiological tools, we may have still missed some true positive cases of TBP. We therefore performed an alternative analysis including definite and probable TB; the latter was defined as a high clinical likelihood of TBP such that TB treatment was administered by the expert clinician presiding over the case (this is likely to provide a more realistic indication of sensitivity). When this approach was used, the sensitivity of IRISA-TB was significantly higher. That the specificity of both tests remained high (95%–100%) even when this approach was used (combination definite and probable TB) suggests that the rate of non-TB cases (false positives) included in this analysis was likely very low.

The sensitivity of Xpert Ultra was limited and similar to that of the Xpert MTB/RIF assay [15]. This is likely because of the paucibacillary nature of the disease and limited antigen levels in serosal fluid below the detection limit, even of the more sensitive Xpert Ultra assay. This is confirmed by the similar cycle threshold values with the older Xpert MTB/RIF and newer Xpert Ultra assays [15]. Would using a higher pericardial fluid volume with centrifugation have increased sensitivity? We previously showed that using a higher volume with centrifugation of the sample failed to increase the sensitivity of the Xpert MTB/RIF assay in TBP [8].

There are some limitations of our findings. This study was conducted in a high TB and HIV setting, which may limit the generalizability of the findings. Nevertheless, TBP is a major problem in sub-Saharan Africa, where HIV coinfection rates are high. Furthermore, we found that IRISA-TB performed even better in persons uninfected by HIV likely because of the more potent and robust Th1 responses at the site of disease in such patients. The sample size of the study was limited, resulting in wider-than-desired sensitivity confidence intervals. Nevertheless, this remains the only study evaluating Xpert Ultra for pericardial TB. A Cochrane meta-analysis published in 2021 that evaluated the Xpert MTB/RIF assay identified just 1 study with >20 participants who were positive for TBP [19]. Finally, we did not use pericardial biopsy, which could have strengthened the reference standard. However, pericardial biopsy is hardly ever used to confirm the diagnosis except in unusual or challenging cases and is inaccessible in most TB-endemic countries. Thus, to strengthen the reference standard, in addition to culture, we used an alternative nucleic acid amplification test to avoid confirmation bias (we could not include Xpert Ultra in the reference standard for this reason). This approach also avoided significant misclassification bias with use of culture only as a reference standard.

In conclusion, despite the higher reported sensitivity of Xpert Ultra when compared with Xpert MTB/RIF in sputum samples, in patients with suspected TBP, the sensitivity and NPV of Xpert Ultra for TB in pericardial fluid were low. By way of comparison, the sensitivity and overall diagnostic performance of unstimulated IFNγ (IRISA-TB) were significantly higher than Xpert Ultra, with excellent accuracy values consistent with those reported in the setting of TBP in a recent meta-analysis [16].

These data inform the selection of diagnostic tests in TB-endemic countries and underscore the precept that in some types of extrapulmonary TB characterized by TB serositis or the accumulation of fluid, host biomarkers outperform traditional microbiological tools. Interestingly, a similar trend is being seen in pulmonary TB where host biomarkers (blood-based gene transcripts) have excellent sensitivity for the diagnosis of pulmonary TB [21] and may be useful, especially in patients with sputum-scarce TB.

## Notes

***Acknowledgments.*** We are indebted to the participants who took part in this study. We thank the health directorate of the City of Cape Town for providing access to appropriate health care facilities. We further acknowledge the assistance of clinical staff at each TB clinic, the University of Cape Town Research Ethics Committee, and community leaders and the University of Cape Town Lung Institute TB community advisory board for enabling this study.

***Patient consent statement.*** Written informed consent was obtained from each patient, and this study was approved by the University of Cape Town Human Research Ethics Committee (HREC REF 370/2015).

***Financial support.*** This work was supported by the South African Medical Research Council through funding received from the South African National Treasury (RFA-EMU-02-2017 to KD; MRC-RFA-UFSP-01-2013 to MN); European and Developing Countries Clinical Trials Partnership (TMA-2015SF-1043 and TMA-1051-TESAII to K. D.); UK Research Initiative (UKRI), Medical Research Council (United Kingdom) (MR/S03563X/1); Wellcome Trust (MR/S027777/1); and European and Developing Countries Clinical Trials Partnership (TMA-2015CDF-1052 to A. E.). The funders have not influenced the results of this research in any way.
